# Is telephone health coaching a useful population health strategy for supporting older people with multimorbidity? An evaluation of reach, effectiveness and cost-effectiveness using a ‘trial within a cohort’

**DOI:** 10.1186/s12916-018-1051-5

**Published:** 2018-05-30

**Authors:** Maria Panagioti, David Reeves, Rachel Meacock, Beth Parkinson, Karina Lovell, Mark Hann, Kelly Howells, Amy Blakemore, Lisa Riste, Peter Coventry, Thomas Blakeman, Mark Sidaway, Peter Bower

**Affiliations:** 1 NIHR School for Primary Care Research, Centre for Primary Care, Manchester Academic Health Science Centre, University of Manchester, Williamson Building, Oxford Road, Manchester, M13 9PL UK; 2 Manchester Centre for Health Economics, Division of Population Health, Health Services Research & Primary Care, Manchester Academic Health Science Centre, University of Manchester, Manchester, M13 9PL UK; 3 School of Nursing, Midwifery & Social Work, University of Manchester, Manchester, M13 9PL UK; 4 Mental Health and Addiction Research Group, Department of Health Sciences and Hull York Medical School, University of York, York, YO10 5DD UK; 5 NIHR Collaboration for Leadership in Applied Health Research and Care – Greater Manchester and Manchester Academic Health Science Centre, University of Manchester, Manchester, M13 9PL UK; 6 Salford Royal NHS Foundation Trust, Stott Lane, Salford, M6 8HD UK

**Keywords:** multimorbidity, older adults, health coaching, depression

## Abstract

**Background:**

Innovative ways of delivering care are needed to improve outcomes for older people with multimorbidity. Health coaching involves ‘a regular series of phone calls between patient and health professional to provide support and encouragement to promote healthy behaviours’. This intervention is promising, but evidence is insufficient to support a wider role in multimorbidity care. We evaluated health coaching in older people with multimorbidity.

**Methods:**

We used the innovative ‘Trials within Cohorts’ design. A cohort was recruited, and a trial was conducted using a ‘patient-centred’ consent model. A randomly selected group within the cohort were offered the intervention and were analysed as the intervention group whether they accepted the offer or not.

The intervention sought to improve the skills of patients with multimorbidity to deal with a range of long-term conditions, through health coaching, social prescribing and low-intensity support for low mood.

**Results:**

We recruited 4377 older people, and 1306 met the eligibility criteria (two or more long-term conditions and moderate ‘patient activation’). We selected 504 for health coaching, and 41% consented. More than 80% of consenters received the defined ‘dose’ of 4+ sessions.

In an intention-to-treat analysis, those selected for health coaching did not improve on any outcome (patient activation, quality of life, depression or self-care) compared to usual care.

We examined health care utilisation using hospital administrative and self-report data. Patients selected for health coaching demonstrated lower levels of emergency care use, but an increase in the use of planned services and higher overall costs, as well as a quality-adjusted life year (QALY) gain. The incremental cost per QALY was £8049, with a 70–79% probability of being cost-effective at conventional levels of willingness to pay.

**Conclusions:**

Health coaching did not lead to significant benefits on the primary measures of patient-reported outcome. This is likely related to relatively low levels of uptake amongst those selected for the intervention. Demonstrating effectiveness in this design is challenging, as it estimates the effect of being selected for treatment, regardless of whether treatment is adopted. We argue that the treatment effect estimated is appropriate for health coaching, a proactive model relevant to many patients in the community, not just those seeking care.

**Trial registration:**

International Standard Randomised Controlled Trial Number (ISRCTN12286422).

**Electronic supplementary material:**

The online version of this article (10.1186/s12916-018-1051-5) contains supplementary material, which is available to authorized users.

## Background

Multimorbidity, defined as ‘the co-existence of two or more chronic conditions, where one is not necessarily more central than the others’ [1], is highly prevalent [2]. Patients with multimorbidity are a major focus of health systems, but they face barriers to accessing high-quality care [3–5], and they incur high costs [6]. Recently, clinical guidelines for multimorbidity have highlighted the need for innovative models of care [7]. Successful self-management will be crucial for improving the health outcomes of patients with multimorbidity, but the current evidence for effectively managing multimorbidity is weak. A recent Cochrane review reported only 18 trials [8], with some evidence for interventions targeted at risk factors such as depression or specific functional difficulties. The review concluded that there is an urgent need for interventions that can help patients with multimorbidity to better self-manage their conditions to prevent exacerbations and avoid expensive care utilisation [9].

For self-management to be cost-effective at a population level, interventions must be delivered to a significant proportion of the population in need, not just those motivated to participate. This is described as ‘reach’ [10]. Evidence of reach is often lacking in trials of self-management, because only a proportion of those meeting the eligibility criteria actually participate [11]. Evidence of reach can be particularly problematic amongst people with multimorbidity because they are often excluded from trials [12]. This study aimed to evaluate the impact of an intervention that can be used with a large number of patients, using a trial design that can better assess the likely population benefit of the intervention.

### The ‘trial within a cohort’ as a test of intervention ‘reach’

In a conventional trial, participants receive information, then provide consent to participate and are randomised. Critically, patients are told about the different treatments available, but only half are randomised to each. Patients with preferences for one treatment may be less likely to take part [13].

The ‘Trials within Cohorts’ (TWiCs) design more closely mimics the way treatment decisions are made in routine care [14]. A cohort of participants are recruited and followed up systematically. Under the form of TWiCs used here, all eligible participants in the cohort are identified, and a sample is *selected at random*. Patients selected for the intervention are contacted and offered the treatment, which they can either decide to receive — and provide informed consent — or decline. Whether or not a patient consents to treatment, for the purposes of this design, they remain part of the intervention arm. All those eligible but not selected are not contacted for participation and become controls.

The TWiCs design has two potential advantages. It more closely mimics the process of treatment decision-making in routine care, as patients are offered a treatment (which they can decline) rather than being offered two treatments, then allocated at chance. The design also provides a different (and in some contexts more useful) estimate of the effects of the *offer of treatment* amongst all those who are eligible, rather than amongst a subset who agree to receive the treatment. As such, it may have greater relevance for treatments designed to have broad ‘reach’ amongst the wider population. Examples would include diabetes prevention programmes [15] and self-management programmes for older people with long-term conditions [16, 17].

### Health coaching as a population health intervention

*S*elf-management is critical for patients with long-term conditions. A model that has received significant attention is health coaching, defined as ‘a regular series of phone calls between patient and health professional...to provide support and encouragement to the patient, and promote healthy behaviours such as treatment control, healthy diet, physical activity and mobility, rehabilitation, and good mental health’ [18].

Various types of health coaching exist that differ in content, delivery (face to face, remote), and personnel. An important issue is whom is targeted for health coaching. It can be provided for patients predicted to be high users of services or following events such as hospital discharge [19]. Although the rationale for such targeting is clear, many patients identified as high users of care revert to lower patterns over time without intervention [20]. There may be an argument for broader strategies targeting the wider population of patients who are currently well but whose current self-management is not optimal. These patients can be described as being less ‘activated’. Patient activation is defined as how well a patient understands his/her own role in personal health care, reflecting knowledge, skills and confidence [21, 22]. Activation may be a method of targeting coaching to maximise benefit. Another important factor may be depression, which is associated with poor outcomes in multimorbidity and may be important in self-management [23]. Treatment burden is an additional factor of relevance in this patient population. It is defined as ‘the impact of the “work of being a patient” on functioning and well-being’ [24, 25] and occurs when the tasks of managing multiple conditions become a detriment to health and well-being.

An increasing number of systematic reviews have been published on the effectiveness of health coaching. Most suggest significant, modest short-term benefits, and some also support longer term gains [26–33]. However, it is difficult to generalise these findings to care for people with multimorbidity, as many trials are focussed on people with only one long-term condition [28, 32]. Further research is indicated to examine the impact of health coaching, assessing reach and the cost-effectiveness of this intervention amongst patients with multimorbidity.

## Methods

### Study design and participants

The study was embedded in a wider integrated care programme to improve care for older people with long-term conditions in North West England. The CLASSIC study is a longitudinal cohort study evaluating this integrated care programme. Embedded within CLASSIC, the Proactive Telephone Coaching and Tailored Support (PROTECTS) trial used the TWiCs design to assess the cost-effectiveness of health coaching for patients with multimorbidity. PROTECTS is reported as per Consolidated Standards of Reporting Trials (CONSORT) guidelines (see Additional file 1: CONSORT checklist). The trial protocol is also included as an additional file (Additional file 2).

The integrated care programme was delivered to patients over the age of 65 with at least one long-term condition, and we recruited these patients to the CLASSIC cohort [34]. FARSITE is a software package (http://nweh.co.uk/products/farsite) that enables centralised searching of general practitioner (GP) records. FARSITE was used to generate a list of eligible patients in each practice, and the results were provided to general practices to allow them to remove any patients meeting the exclusion criteria (patients in palliative care or with reduced capacity to consent) prior to asking them for consent. A total of 12,989 patients were eligible between November 2014 and February 2015. If they did not respond, they were sent a reminder 3 weeks later. Participants were offered an incentive of a £10 voucher. At baseline, 4377 people (34.2%) returned a questionnaire. We did not have access to data on non-respondents.

For inclusion in PROTECTS, patients had to have 2 or more self-reported long-term conditions from a list of 15 [35], and must have been assessed as needing some assistance with self-management, defined via scores on the Patient Activation Measure (PAM) [36]. The PAM allows activation to be categorised into four levels. Level 1 includes passive recipients of care, level 2 includes those who lack the basic knowledge and confidence to self-manage, level 3 is those who have the basic knowledge but lack the confidence and skills to engage in self-management and level 4 is those who have the knowledge, confidence and skills and may only require support during times of stress [36]. We included patients in PROTECTS whose scores placed them in level 2 or 3 of activation, because these patients showed some evidence of self-management which could be improved by health coaching.

#### Randomisation and masking

As noted earlier, patients eligible for the trial are identified from the cohort and randomly selected for treatment. We piloted these procedures in 50 patients to test the rate of uptake of the new treatment. After assessment of eligibility, we selected patients to be offered health coaching at random, using appropriate central randomisation through a clinical trials unit to ensure concealment of allocation. In this pragmatic evaluation, we did not blind either patients or providers.

#### Procedures

The intervention was health coaching, as defined earlier. The content of the health coaching was based on three core mechanisms:

1. *Telephone health coaching* involved support and encouragement to the patient to promote healthy behaviours around diet, exercise, smoking and alcohol, through provision of information and motivation for long-term conditions. The core health coaching materials include telephone and associated patient tracking and management software, and health coaching scripts for lifestyle support.
2. *Social prescribing* involved links to resources in the wider community through the community and voluntary sector [37, 38]. Access to local resources was provided through either PLANS (http://www.plansforyourhealth.org/, a self-assessment tool for users to assess their health and social needs, with links to relevant community resources and local support) or the Ways to Well-being site (on-line resources and information, no longer available in the form used in the trial).
3. *Low-intensity support for low mood* included assessment of common mental health problems, simple lifestyle advice and behavioural techniques to manage mood, and use of appropriate risk assessment protocols [39, 40].

Six monthly phone calls to participants were planned. The receipt of four out of the six planned calls was considered a complete ‘dose’ of the intervention.

The PROTECTS intervention was delivered by a ‘health advisor’ (a National Health Service (NHS) Agenda for Change Band 4 worker) with skills in information technology and communication, as well as experience in working with the general public. Advisors already had experience with coaching for diabetes and use of social prescribing. The health advisor attended 3 days of training specific to working with low mood. They were given a manual which outlined the key elements of the low-intensity intervention used (behavioural activation, cognitive restructuring, problem solving). They also received monthly group clinical supervision which focussed on working with low mood. The health advisor were further supported by a specialist nurse manager and received additional advice on mental health and social prescribing (i.e. referral to relevant community resources) from the research team. Patients routinely had continuity in their coach for the duration of their treatment. There were no formal links with primary care as part of the intervention. The health coaching was delivered via telephone from a central NHS facility. Proactive, monthly calls of around 20 min were made for a period of 6 months, with the option for additional calls to deal with complex patients or issues of risk. Health coaching staff were trained to customize calls to the individual patient. Provision of support for low mood and social prescribing were made where appropriate.

The design meant that the comparator for patients meeting the eligibility criteria who were not selected for the intervention was usual NHS care. We collected details of that care for the economic evaluation.

#### Outcomes

PROTECTS was nested within the CLASSIC cohort, which used a wide range of measures, varying at different time points. A pre-specified subgroup of primary outcomes were used in PROTECTS. All outcomes were collected via postal survey at four time points across the study: at baseline, then at 6, 12 and 20 months. The protocol was registered and updated in a registry (ISRCTN 12286422).

The primary outcome measures were:

*- Self-management.* The PAM is a self-report measure of patient knowledge, skills and confidence in self-management for long-term conditions [22, 36, 41]. We used the short 13-item version. The score is categorised into four levels for eligibility determination, although we used the continuous score in the analyses.
*- Quality of life.* The World Health Organization Quality of Life brief measure (WHOQOL-BREF) is a 26-item measure of global quality of life (QOL), which has been validated in a large international population with physical and mental long-term conditions. QOL is measured across four domains: physical, psychological, social and environmental, as well as a single-item scale for QOL [42]. We used the physical domain score as the most relevant in relation to the PROTECTS intervention.

Secondary outcome measures were:

*- Depression.* The Mental Health Inventory (MHI-5) is a 5-item scale which measures general mental health [43]. This measure is well validated for identifying depression symptoms, with a higher score indicating better mental health [44, 45]. The recommended cutoff score of 60 was used to indicate the presence of ‘probable depression’ [45], although we used the continuous score in the analyses.
*- Self-care.* The Summary of Diabetes Self-Care Activities (SDSCA) is a 7-item measure assessing the number of days per week respondents engage in healthy and unhealthy behaviours (i.e. eating fruits and vegetables, eating red meat, undertaking exercise, drinking alcohol and smoking) [46].

#### Power and statistical analysis

At the time of study development, there were no bespoke methods for powering this TWiCs design, and we used conventional methods [47]. We powered the study to have 80% power (alpha 5%) to detect a standardised effect size of 0.25 on any continuous outcome measure. Allowing for 25% attrition amongst participants — and assuming that outcome measures at baseline correlate 0.5 with their respective follow-ups — 504 patients were indicated, with 252 randomised to treatment. The CLASSIC cohort included 1306 patients eligible for PROTECTS, and we randomly selected 252 to be offered the intervention. The uptake rate was lower than anticipated, and we therefore offered the intervention to a further 252 patients. This resulted in a final intervention group of 504 of which 207 consented to the intervention, with the remaining 802 as controls. However, under the TWiCs framework, all 504 patients offered treatment remain in the treatment group in analysis, including those who declined. In consequence, the eventual effect size detectable at 80% power was 0.39 amongst the subsample consenting to treatment.

The analysis followed intention-to-treat principles and a pre-specified analysis plan. In summary, we report the trial and analysis according to updated CONSORT standards and utilising the extension for pragmatic trials [48]. The main hypothesis test of the intervention was that the overall effect of the intervention is zero. The primary analysis used complete cases only. Condition group was used as a binary variable. All outcomes were treated as though continuous and normally distributed (in all cases both skewness and kurtosis were < =1.0) and analysed using linear multiple regression. Baseline values of outcomes and a set of pre-specified covariates considered prognostic of outcome were included in all analyses: gender, age (categorised as 65–69, 0–79, 80–98), health literacy [49], social support [50], patient activation, depression and quality of life (physical health domain). Robust estimates of variance were used accounting for the clustering of patients within practices.

We ran two sensitivity analyses. The first repeated the primary analyses using multiple imputation to include cases with missing baseline or follow-up data. Missing data values were imputed using chained-equation multiple imputation and scores on all available outcome measures and patient demographics at baseline and follow-up. Twenty multiple imputation sets were used to ensure stability of results. The second sensitivity analysis assessed the robustness of the primary analysis results to removal of the pre-specified covariates from the model (not including the outcome at baseline).

Health coaching in the trial was delivered by an existing service managing other patients outside the trial, rather than a bespoke service. This, combined with the time taken to administer and analyse the cohort and randomly select the groups, meant that no patient was offered treatment until 6 months after the baseline assessment for the CLASSIC cohort, and for some the offer was not made until month 12 or later. This caused variations in the duration of time before start of the treatment (range 259 to 513 days after baseline assessment). Length of follow-up from end of treatment to 20 months follow-up was similarly variable. Thus, the trial is considered to have run over 20 months, with patients receiving treatment at any time after the initial 6 months. As these implementation delays were not anticipated, the pre-specified analysis plan stated that the primary analysis would assess the change in outcomes between baseline and 20 months follow-up.

The design provides an estimate of the mean effect in people offered treatment. Compared to a pragmatic trial, which provides an estimate of the mean effect in people agreeing to treatment, the effect is ‘diluted’ by the proportion of patients in the treatment arm who do not consent to treatment. An estimate of the treatment effect in those patients consenting to treatment was derived through application of a complier average causal effect (CACE) analysis [51, 52]. The CACE estimator was obtained by dividing the mean effect estimate by the proportion giving consent [51]. The CACE estimate is typically larger, but the power to detect an effect is not greater, since the variance of the estimate increases proportionately [53].

#### Cost-effectiveness analysis

The primary outcome measure for the economic evaluation was the EuroQOL 5-Dimension 5-Level (EQ-5D-5L) [54], a generic measure of health-related QOL covering five domains (mobility, self-care, usual activities, pain/discomfort, anxiety/depression). This new version was developed due to concerns over the lack of sensitivity to change of the original scale, and consists of five severity levels for each domain. Published English general population preference weightings were used to convert responses to a single utility index [55].

The perspective of the economic analysis was that of the English NHS. Individual patient-level health care resource utilisation over the trial period was collected from two sources. The number of GP contacts in the previous 6 months was collected from self-report data at 6-monthly intervals. Hospital utilisation was extracted from linked administrative patient records provided by the NHS, divided into emergency admissions (short stays ≤5, long stays > 5 days), elective admissions, elective day cases, outpatient attendances and accident and emergency (A&E) department attendances.

The economic analysis assessed the incremental cost-effectiveness of the offer of health coaching compared with usual care from the perspective of the NHS. EQ-5D-5L data were combined with in-hospital mortality information from the secondary care utilisation data, applying a utility value of 0 upon death. Quality-adjusted life years (QALYs) were calculated using the area under the curve method assuming linear extrapolation of utility between time points. QALYs in the second year of the trial were discounted at an annual rate of 3.5% as specified by NICE [56].

Intervention costs were estimated combining the cost of training and supervision, written materials and delivery of the health coaching sessions. The intervention was offered to all participants selected, although only 189 received at least one call. Only patients receiving at least one call were assigned treatment costs, and the intervention costs were therefore estimated based on these 189 participants.

Patient-level resource utilisation data were combined with relevant unit cost data for the price year 2014–2015 to calculate total costs. Unit costs not available for this price year were inflated to 2014/2015 prices using the consumer price index [57]. Costs occurring in the second year were discounted at a rate of 3.5% [56]. Unit cost figures were sourced from the Personal Social Services Research Unit’s unit costs of Health and Social Care 2015 and national NHS Reference Costs [58, 59].

Follow-up questionnaire completion dates were missing in a small number of cases (*n* = 2). In these instances, dates were imputed using the mean length of time between baseline and follow-up for the sample for the purpose of QALY and cost calculations. Missing information on age and gender were sourced from the linked hospital administrative data, where available (gender *n* = 6, age *n* = 35). For the remaining individuals with missing age (*n* = 30) or missing baseline EQ-5D-5L (*n* = 29), mean imputation was used to ensure independence from treatment allocation [60].

For missing EQ-5D-5L and resource use data, we used multiple imputation by chained equations (ICE) to generate 50 imputed datasets assuming the data were missing at random. The independent variables specified in the imputation models were age, gender, treatment arm and baseline EQ-5D-5L. To account for non-normality, predictive mean matching was used which forces imputations to only take values observed in the original dataset. Multiple imputation (MI) was conducted using Stata’s ICE package, and analysis using Stata’s MI package.

The incremental cost-effectiveness ratio (ICER) was calculated, adjusting for age, gender, and baseline EQ-5D-5L index score [61]. To assess uncertainty surrounding the estimates and to account for the typically skewed nature of cost data, incremental costs and QALYs were bootstrapped using pairwise bootstrapping with replacement using 10,000 replications. Cost-effectiveness planes plot these 10,000 bootstrap replications of the ICER estimates to illustrate the uncertainty around the point estimate of the ICER in probabilistic terms. Finally, cost-effectiveness acceptability curves (CEACs) were plotted to graphically represent the probability of the intervention being cost-effective across a range of cost-effectiveness thresholds.

The primary economic analysis was based on a comparison on the full sample with MI. A sensitivity analysis was performed using only the complete case sample for which there were no missing data. We also took advantage of the implementation delays to perform a further sensitivity analysis separating the trial period into two parts: baseline to 6 months follow-up, where no treatment had yet been received; and 6 months to 20 months follow-up, where we expect any treatment effects to occur. Stata version 14 was used in the analysis.

## Results

### Recruitment, retention and baseline characteristics

**Fig. 1:**
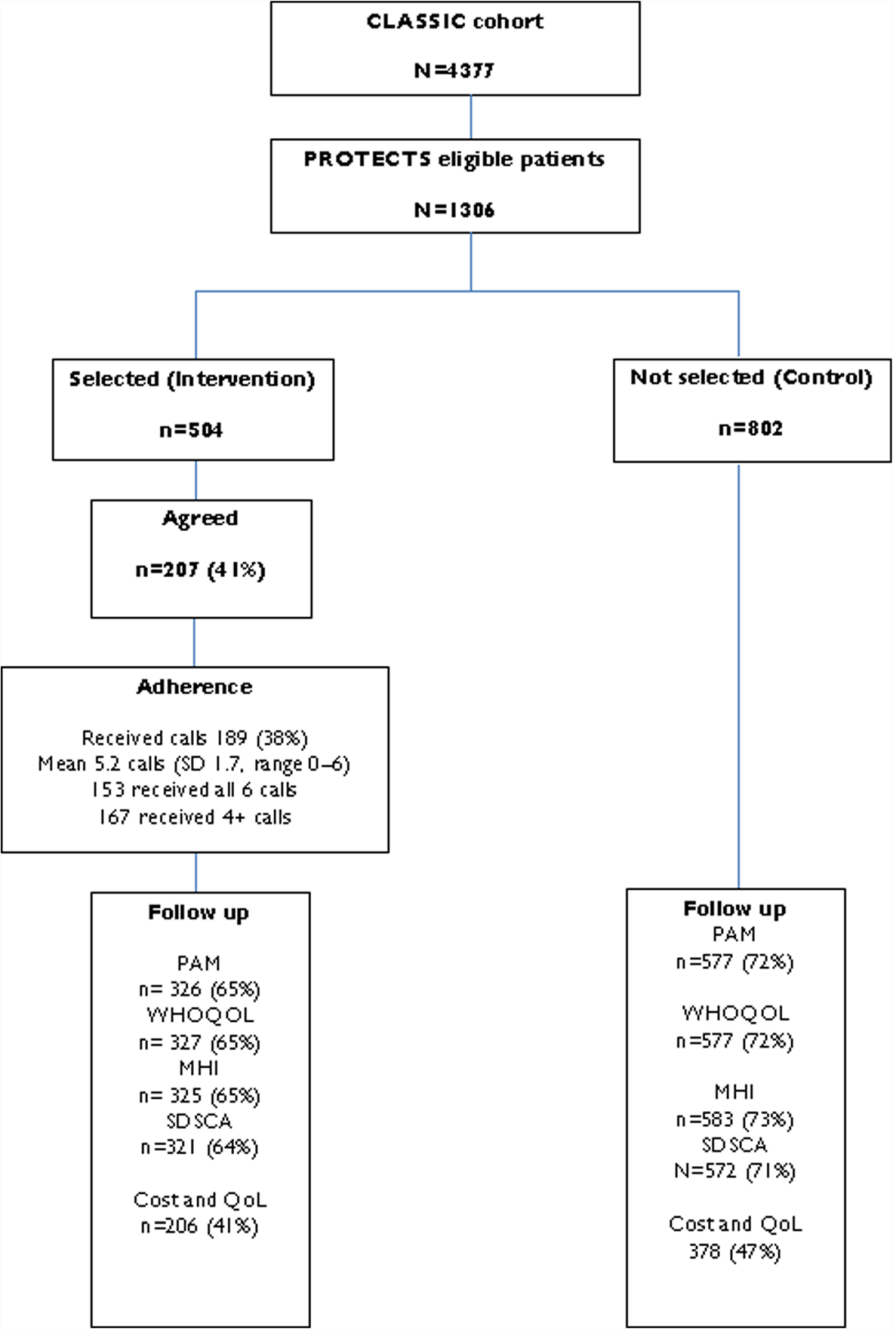
PROTECTS CONSORT diagram

In total, 12,989 patients were identified as eligible for the cohort, and at baseline 4377 (33.6%) participated. Of those, 1306 were eligible for PROTECTS. Of the 1306, 504 were randomly selected to the intervention, and the remaining 802 eligible participants acted as controls. The flow of participants is shown in Fig. 1. The baseline characteristics of participants are presented in Table 1.

**Table 1:** Baseline characteristics of participants

Characteristics	Not selected (*n* = 802)	Selected (*n* = 504)	Total (*n* = 1306)
Mean (SD) age	74.2 (6.4)	75.4 (6.8)	74.7 (6.6)
Age in categories:			
65–69 years	216 (26.9)	115 (22.8)	331 (25.3)
70–79 years	385 (48.0)	230 (45.6)	615 (47.1)
80–98 years	155 (19.3)	140 (27.8)	295 (22.6)
Sex (%):			
Female	441 (55.0)	270 (53.6)	711 (54.4)
Male	357 (44.5)	232 (46.0)	589 (45.1)
Health literacy:			
Never	536 (66.8)	322 (63.9)	858 (65.7)
Rarely	100 (12.5)	57 (11.3)	157 (12.0)
Sometimes	87 (10.9)	63 (12.5)	150 (11.5)
Often/always	59 (7.4)	44 (8.7)	103 (7.9)
Living status (%):			
Live with partner or others	509 (63.5)	315 (62.5)	824 (63.1)
Live alone	288 (35.9)	188 (37.3)	476 (36.5)
Education (%):			
No qualifications	352 (43.9)	221 (43.9)	573 (43.9)
School level qualifications	68 (8.5)	56 (11.1)	124 (9.5)
College degree or higher	349 (43.5)	191 (37.9)	540 (41.4)
Mean (SD) chronic conditions	6.8 (2.6)	6.8 (2.5)	6.8 (2.6)
Mean (SD) index of multiple deprivation	31.0 (18.8)	33.0 (18.6)	31.8 (18.7)
Employment (%):			
Retired or not economically active	748 (93.3)	472 (93.7)	1220 (93.4)
Working or other	39 (4.7)	23 (4.6)	62 (4.8)
Ethnicity (%):			
White	786 (98.0)	489 (97.0)	1275 (97.6)
Non-white	11 (1.37)	12 (2.4)	23 (1.8)
Mean (SD) GP visits in past 6 months	3.1 (2.0)	3.0 (1.9)	3.1 (1.9)
Mean (SD) patient activation	57.8 (6.0)	57.6 (5.6)	57.8 (5.9)
Mean (SD) quality of life (physical health)	55.3 (19.8)	54.0 (18.8)	54.8 (19.4)
Mean (SD) depressive symptoms	65.3 (21.3)	65.3 (21.8)	65.3 (21.3)
Possible depression diagnosis (%):			
Depression	371 (46.3)	227 (45.0)	598 (45.8)
No depression	426 (53.1)	265 (52.9)	691 (52.9)
Mean (SD) self-care activities	3.8 (0.9)	3.8 (0.9)	3.8 (0.9)

### Treatment uptake and adherence

Signed consent to health coaching amongst those eligible was received from 207/504 (41%) of those selected, although only 189 actually received calls (38%). The baseline characteristics of consenters and non-consenters are reported in Additional file 3: Table A. A multivariate logistic regression exploring baseline factors associated with consent found that only younger age (odds ratio (OR) = 1.08, 95% confidence interval (CI) = 1.03–1.14) and higher education (OR = 4.07, 95% CI = 2.08–7.94) predicted consent to health coaching.

Among those who consented, 167/189 (85%) received 4+ calls (the predefined ‘dose’). Assessment of call content showed that diet and exercise were the most common areas dealt with (in 70% and 57% of patients respectively), whereas 25% of patients received social prescribing and around 23% received support for low mood.

### Outcomes

Table 2 shows the patient-reported outcomes for patients selected for the offer of health coaching and those not selected. The adjusted mean differences were small for all of the primary and secondary outcome measures and did not reach statistical significance (*p* > 0.05). The non-significance of all group differences was confirmed in both sensitivity analyses.

**Table 2:** Intention-to-treat analyses of primary and secondary outcomes, using complete cases

	Intervention group (eligible patients selected for treatment)		Control group (eligible patients not selected for treatment)		Comparison		CACE estimates (estimated points change in those consenting to treatment)
	*N*	Mean (SD)	*N*	Mean (SD)	Adjusted difference	*p* value	Adjusted difference in means^a^ (95% CI)
					in means^a^ (95% CI)		
Primary outcomes							
Patient Activation Measure (PAM)	326	62.88 (14.39)	577	61.92 (13.24)	1.44 (−0.46 to 3.33)	0.133	3.69 (−1.17 to 8.53)
WHO Quality of Life —- physical health (WHOQOL)	327	55.74 (19.15)	577	55.41 (18.72)	1.62 (−0.32 to 3.56)	0.099	4.15 (−0.82 to 9.12)
Secondary outcomes							
Depression (Mental Health Inventory, MHI-5)	325	75.74 (16.40)	583	74.29 (17.26)	1.00 (−1.25 to 3.26)	0.373	2.56 (−3.20 to 8.36)
Self-care (SDSCA)	321	3.49 (1.09)	572	3.54 (1.10)	−0.04 (−0.19 to 0.11)	0.58	−0.10 (−0.49 to 0.28)

^a^Adjusted for covariates gender, age, health literacy, social support, patient activation, depression and quality of life

Using CACE analysis, the estimated treatment effects on participants who took up the intervention were higher, but with correspondingly wider non-significant confidence intervals (Table 2).

### Economic analysis

Complete data necessary for the economic analysis were available for 45% of the sample (584/1306).

Table 3 shows EQ-5D-5L utility scores at each time point and the total QALY gain over 18 months for the complete case sample. Patients selected for the offer of health coaching reported slightly lower EQ-5D-5L scores at baseline. This steadily fell at each time point for the usual care group (0.664 at 18 months follow-up), whilst remaining stable for the health coaching group (0.691). The mean unadjusted QALYs for usual care were 1.105, and 1.124 for health coaching over the study period.

**Table 3:** HRQOL outcomes (EQ-5D-5L) amongst the complete case sample

	Usual care (*n* = 378)				Health coaching (*n* = 206)			
	Mean	SD	Min	Max	Mean	SD	Min	Max
Baseline	0.708	0.23	−0.18	1	0.696	0.236	−0.102	1
6 months	0.691	0.247	−0.185	1	0.709	0.228	0.018	1
12 months	0.685	0.254	−0.246	1	0.694	0.237	0	1
18 months	0.664	0.264	−0.18	1	0.691	0.26	0	1
QALYs	1.105	0.374	−0.29	1.723	1.124	0.355	0.055	1.683

The resources required to deliver the health coaching intervention are presented in Additional file 3: Table B. The average cost per individual receiving the full course of health coaching (6 calls) was £148.27. In addition to the direct costs, the analysis also considered the wider NHS resource utilisation. Table 4 reports the average utilisation by resource category for the complete case sample. Overall, there was a pattern of greater use of emergency care amongst the control group, whilst the group offered health coaching used more planned services.

**Table 4:** Resource utilisation amongst the complete case sample

	Baseline to 6 months	
Type of service	Usual care (*n* = 378)	Health coaching (*n* = 206)
	Mean (95% CI)	Mean (95% CI)
Secondary care contacts		
Emergency short stay	0.063 (0.039—0.088)	0.058 (0.026–0.091)
Emergency long stay	0.026 (0.009–0.044)	0.024 (0.003–0.045)
Day case	0.172 (0.104–0.240)	0.112 (0.059–0.165)
Elective admission	0.024 (0.008–0.039)	0.029 (0.002–0.056)
Outpatient	4.992 (4.162–5.823)	6.553 (4.977–8.130)
A&E attendance	0.156 (0.110–0.203)	0.131 (0.083–0.179)
GP appointments	3.111 (2.791–3.431)	3.039 (2.641–3.437)
	6 months to 12 months	
Secondary care contacts	Mean (95% CI)	Mean (95% CI)
Emergency short stay	0.050 (0.027–0.074)	0.039 (0.006–0.072)
Emergency long stay	0.040 (0.010–0.069)	0.019 (0.000–0.038)
Day case	0.127 (0.069–0.185)	0.053 (0.017–0.090)
Elective admission	0.029 (0.009–0.049)	0.029 (0.002–0.056)
Outpatient	4.595 (3.650–5.540)	6.403 (5.126–7.680)
A&E attendance	0.159 (0.108–0.209)	0.097 (0.041–0.153)
GP appointments	2.783 (2.527–3.039)	3.058 (2.696–3.421)
	12 months to 18 months	
Secondary care contacts	Mean (95% CI)	Mean (95% CI)
Emergency short stay	0.132 (0.091–0.174)	0.068 (0.028–0.108)
Emergency long stay	0.045 (0.022–0.068)	0.034 (0.009–0.059)
Day case	0.196 (0.107–0.284)	0.180 (0.105–0.254)
Elective admission	0.040 (0.020–0.059)	0.063 (0.027–0.099)
Outpatient	7.185 (6.064–8.307)	9.893 (8.570–11.217)
A&E attendance	0.275 (0.207–0.343)	0.170 (0.112–0.228)
GP appointments	2.865 (2.599–3.131)	2.922 (2.543–3.302)

Table 5 presents the average costs of the resource utilisation of the complete case sample. The list of unit costs and resources is available in Additional file 3: Table C. The most costly category was outpatient appointments, followed by elective admissions and GP appointments. These are all planned care services, the costs of which were higher in the health coaching group. Conversely, the costs of emergency admissions (short and long stays), day cases, and A&E attendances were higher in usual care. Overall, mean costs were higher in health coaching (£4000.88) than usual care (£3424.16). The average intervention costs in health coaching were £79.29. This is lower than the £148.27 estimated for a course of health coaching because not all individuals took up or completed the health coaching.

**Table 5:** Resource use costs amongst the complete case sample

Type of service	Usual care (*n* = 378)	Health coaching (*n* = 206)
	Mean (£) 95% CI	Mean (£) 95% CI
Secondary care costs		
Emergency short stay	146.87 (112.25–181.48)	98.95 (64.27–133.63)
Emergency long stay	313.76 (190.97–436.54)	219.08 (101.92–336.24)
Day case	343.61 (212.29–474.93)	238.36 (166.87–309.86)
Elective admission	310.71 (203.04–418.38)	405.96 (201.93–609.99)
Outpatient appointment	1851.42 (1605.13–2097.70)	2521.95 (2139.57–2904.32)
A&E attendance	76.66 (62.69–90.63)	51.79 (39.33–64.24)
Mean total costs of secondary care contacts	3043.02 (2626.02–3460.03)	3536.09 (2979.87–4092.31)
GP appointments	381.14 (350.96–411.32)	392.50 (351.72–433.28)
Health coaching costs	–	79.29 (69.59–88.99)
Mean total cost	3424.16 (2999.98–3848.34)	4007.88 (3444.57–4571.18)

### Cost-effectiveness analysis: full sample with imputation

Table 6 presents the adjusted estimates of the effects of the offer of health coaching on the incremental costs and QALYs compared to usual care in the full sample with imputed data, controlling for age, gender and baseline utility.

**Table 6:** Cost-effectiveness analysis: full sample with imputation

Health coaching (*n* = 504) over usual care (*n* = 802)	Mean	Bootstrapped standard error	Bootstrapped 95% CI	
Incremental cost (£)	150.583	316.941	−470.611	771.776
Incremental QALYs	0.019	0.012	−0.006	0.043
ICER	£8049.96			

The offer of health coaching is associated with a mean incremental total cost increase of £150.58 (95% CI £–470.611, £711.776) and a mean incremental QALY gain of 0.019 (95% CI –0.006, 0.043).

**Fig. 2:**
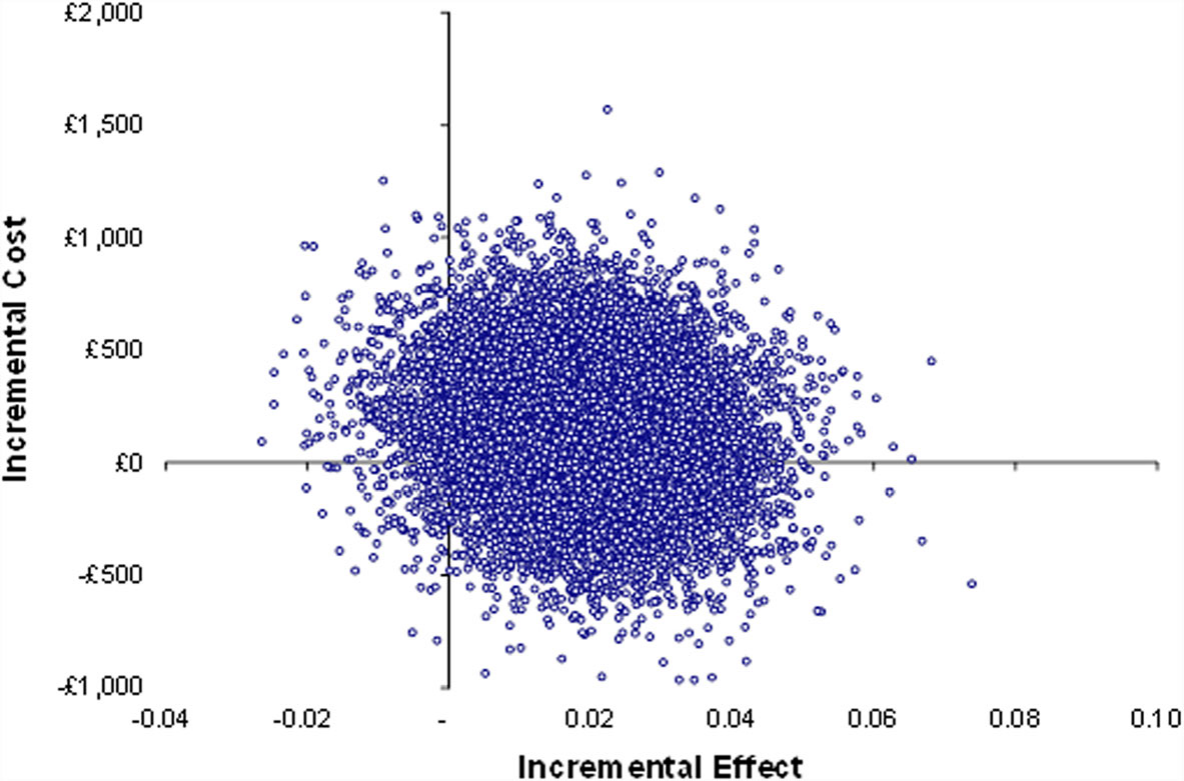
Cost-effectiveness plane: full sample with imputed data

Whilst there are no statistically significant differences in either costs or QALYs, the point estimate of the ICER is £8049.96 per QALY. This would represent a cost-effective intervention at the standard cost-per-QALY threshold of £20,000–30,000. However, it is important to consider the uncertainty surrounding this estimate. The cost-effectiveness plane plots the 10,000 bootstrap replications of incremental cost and QALY estimates (Fig. 2). The replications are clustered in the north-east quadrant in Fig. 2 (positive health gain and increased cost). Health coaching resulted in an incremental QALY gain in 94% of bootstrap replications and was higher cost in 69% of replications.

**Fig. 3:**
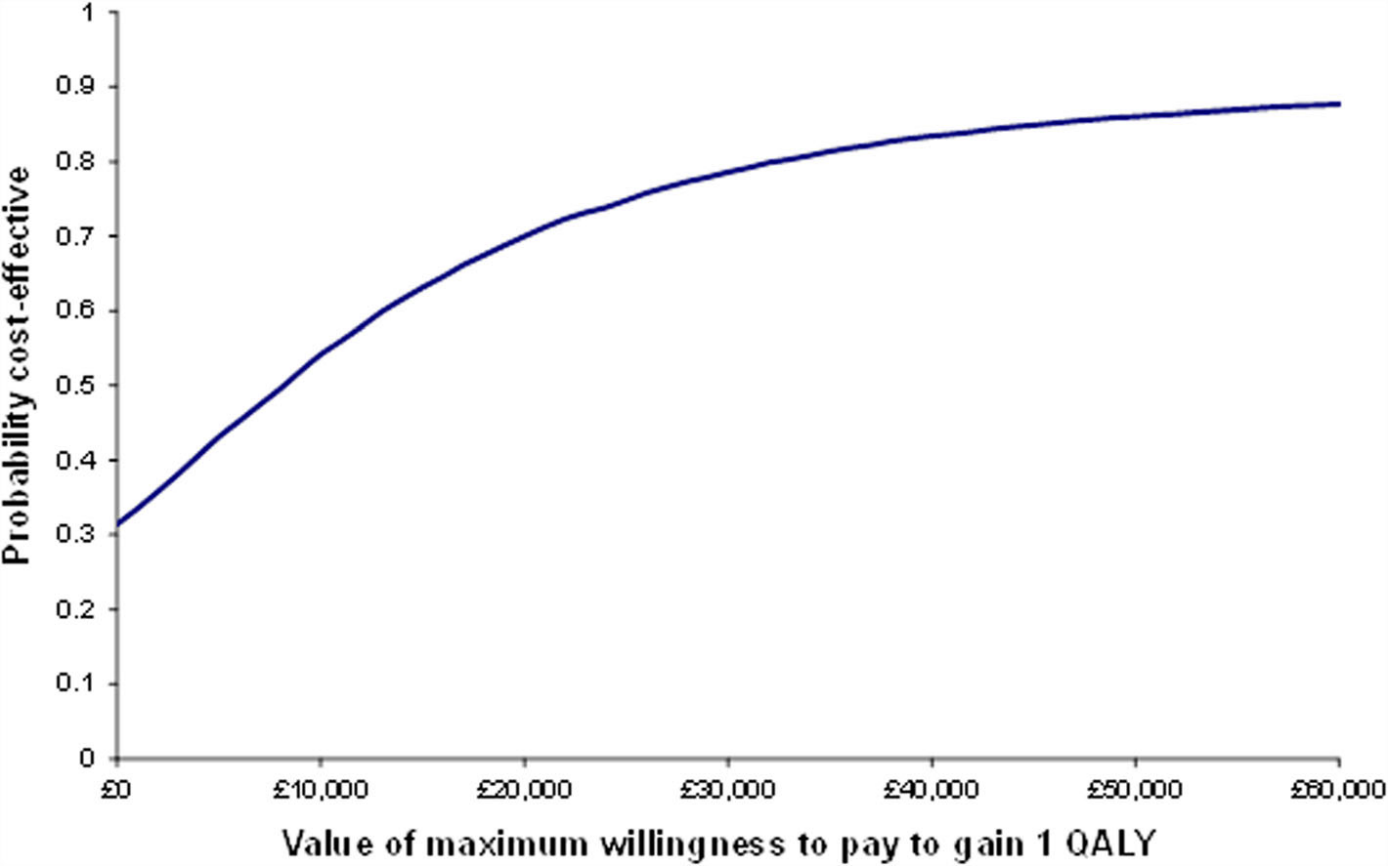
Cost-effectiveness acceptability curve: full sample with imputed data

The CEAC (Fig. 3) demonstrates how the probability that health coaching is cost-effective increases with the decision-maker’s willingness to pay. At the lower bound threshold of £20,000 per QALY, there is a 70% probability of health coaching being cost-effective. This rises to 79% at the upper bound of £30,000. Compared with usual care, health coaching is likely to be cost-effective in 50% or more cases if decision-makers are willing to pay £8180 or more for a QALY.

The results of the cost-effectiveness analyses were similar when a complete case analysis was undertaken (see Additional file 4). The post hoc sensitivity analysis analysing costs and outcomes separately in the first 6 months post baseline (when no health coaching was received) confirmed that the period in which participants actually received treatment was driving outcomes, as the effects were restricted to the period in which health coaching was delivered (see Figures C to F in Additional file 4).

## Discussion

### Principal outcomes

We evaluated the role of health coaching in the care of multimorbidity. We showed reasonable levels of intervention uptake amongst older patients with multimorbidity who were not actively seeking help with self-management. A large proportion of those who accepted the referral to health coaching received a defined ‘dose’. Assistance with diet and exercise were the most common interventions within health coaching, although support for low mood and social prescribing were also present for a significant minority.

Analysis of health outcomes demonstrated no significant benefit associated with health coaching. However, the economic analysis suggested that health coaching resulted in an incremental increase in both costs and QALYs. When a QALY was valued at £20,000, there was a 70% probability that health coaching was cost-effective. The economic analysis suggested that health coaching led to higher utilisation of planned services and lower use of emergency hospital services than usual care.

### Strengths and limitations

In addition to its large size and focus on multimorbidity, this trial employed the novel ‘Trials within Cohorts’ design. This design provides evidence of ‘reach’ because it assesses uptake amongst people not actively seeking treatment. A major criticism of conventional trials is that they show effectiveness of an innovation in a very selected group of patients, which then fails to ‘scale’ because of issues such as low rates of acceptability amongst the wider population, and differences between those who take part in trials and those eligible for the intervention [11].

However, this trial also has important limitations, some of which are directly associated with the TWiCs design. A conventional pragmatic trial assesses intervention effects on those consenting to treatment, with an assumption that there will be non-adherence amongst consenters which will reduce any intervention effect (as these are included in any intention-to-treat analysis). The current design estimates the mean effect of selection for treatment, and again all patients selected for treatment must remain in that group in the intention-to-treat analysis. The proportion of selected patients who do not take up the intervention in a ‘trial within a cohort’ will likely always be larger than the proportion of consenting patients who do not comply with treatment in a conventional pragmatic trial. In consequence, the inclusion in the PROTECTS treatment group of 59% of participants selected for the intervention who did not take it up — including 10% who were uncontactable — greatly diluted the overall treatment effect compared to controls, and resulted in a detectable standardised effect (amongst those consenting to treatment) of 0.39, rather than the 0.25 initially powered for. We have since published specific methods for estimating sample sizes for this type of design [47].

Our ability to detect an effect is likely to have been further reduced by the use of data collected at fixed time intervals, as start of treatment varied greatly relative to the collection of baseline measures — with correspondingly wide variation between end of treatment and 20 months follow-up. The logistics of the research and capacity within the service meant that no participant was offered the intervention prior to the 6 months follow-up. Changes in health or behaviours over this period may have an impact on the effectiveness of an intervention, possibly reducing differences between groups. Nevertheless, delays in accessing treatment are common in routine service delivery. Another ‘trial within a cohort’ (the Depression in South Yorkshire (DEPSY) trial) achieved a somewhat higher consent rate of 51%, but with 19% of those selected uncontactable [62]. DEPSY experienced a much higher attrition rate in the treatment arm, 32% compared to 13% of controls, and we found some evidence for differential attrition. These and other TWiCs design-related issues are considered in a related publication [47].

The trial cannot answer the question of whether health coaching is effective and cost-effective for multimorbidity in the longer term. The health coaching intervention consisted of three mechanisms, but the design does not allow us to estimate their distinct contribution. Nearly half of the patients reported symptoms of depression, and although support for low mood was provided frequently, it may have to be a more significant aspect of interventions in patients with multimorbidity [63]. The economic analysis was based on 45% of patients who returned complete data, which may limit the general conclusions. Although multiple imputation was used to impute missing data values, this cannot fully adjust for unmeasured factors that may affect both outcomes and questionnaire completion; hence, the cost-effectiveness findings may be subject to residual confounding. However, a sensitivity analysis comparing cost-effectiveness in the 6 months prior to the intervention — in which time the majority of attrition occurred — with cost-effectiveness under the intervention found the effects restricted to the latter period.

Finally, this trial was conducted amongst patients with multimorbidity in one area in the UK primarily composed of white patients. Ethnic minority groups report poorer experience of care [64], and we do not know whether the effectiveness, reach and cost-effectiveness of health coaching are different in ethnic minority groups with multimorbidity. Although we have described this as a population health approach, we did restrict to certain groups depending on baseline activation, so ‘reach’ was somewhat limited by design. The response rate of patients to the initial cohort recruitment was in line with previous studies in this area [65, 66], but is potentially another source of bias, and with very limited demographic data on non-responders to the initial cohort, we were unable to assess overall representativeness. Although patient inclusion in the cohort was based on data within clinical records, patients self-reported types of long-term conditions, and these were not validated against clinical diagnosis.

### Interpretation of the results in the context of the wider literature

It was felt that this design was a relevant test of health coaching as a population health strategy, reaching out to patients assessed as in need, but who may not necessarily be seeking self-management support. There will naturally be interest in the effects on those patients who engaged. Although per-protocol analyses can be used, such an approach is vulnerable to bias. Some published trials have assessed the effects through propensity matching of the subset who engaged [67]. The CACE analysis is the preferred model for assessment of effects in those who receive the intervention, as under certain, though usually reasonable, assumptions it provides an unbiased estimate of effect.

Further development of the intervention may have to consider different approaches to targeting, or more choice around the exact nature of the intervention to better align with patient preferences. Qualitative research conducted alongside the trial will be published in the full study report and may provide insights into these issues [68]. The group entering the trial did report significant numbers of conditions, and it is possible that they were too ill to benefit from the intervention. As noted earlier, existing treatment burden may be high in these patients, and although the coaching is designed to support self-management, it is possible that adding more self-management may exacerbate issues in treatment burden [69]. Our model of using activation to target the intervention is in line with the suggested uses of the measure [21] and reflects previous health coaching studies which have suggested the importance of avoiding patients who are too ill or too well to benefit [70]. There is good evidence that activation predicts many outcomes, but the evidence that activation can predict differential benefit from interventions is not as strong [34].

The pattern of health utilisation shown in the different groups is of interest. Many interventions for older people target those who demonstrate high levels of health care utilisation, on the basis that this is where reductions are most likely to be made. Nevertheless, it can be difficult to reduce utilisation in such patients in a comparative study [19], as patients identified on the basis of high use may demonstrate regression to the mean, may not be particularly amenable to intervention and may be present in small numbers in the population [20]. One of the largest trials of health coaching undertaken used a risk prediction score for inclusion in the trial, but it failed to demonstrate overall benefits in terms of admission rates [67]. The approach taken in PROTECTS was different, as patients were identified on the basis of showing capacity for improvement in activation. Such patients are prevalent, and the results suggested that the intervention might reduce emergency use of care. However, the positive impacts of such change were ameliorated by increases in elective use and overall increases in costs. Another very large trial of health coaching which showed reductions in costs had an additional focus on ‘preference sensitive’ shared decision-making rather than self-management alone [70].

As noted earlier, the recent Cochrane review reported only limited evidence for patients with multimorbidity [8], although there was a suggestion that interventions targeted at risk factors such as depression or specific functional difficulties might be more effective. Whilst our intervention had a depression component, it was not the primary focus as in other interventions in multimorbidity [63], and it is possible that the broad focus on self-management behaviour change is less impactful than a specific focus on a single area such as depression, especially in the context of an intervention of limited duration. Alternatively, our focus on depression may have paid insufficient attention to other psychosocial issues that might be present in these patients, such as anxiety or functional disorders. It is equally possible that for patients with fairly high levels of multimorbidity, the dose of the coaching was simply insufficient [67]. A longer treatment might have increased effectiveness, although with restricted resources, increasing the length of treatment will clearly restrict ‘reach’.

## Conclusions

Patients with multimorbidity are a major part of the workload of health systems, and findings from large evaluations of new models of care for this patient group are directly relevant to clinicians and policy decision-makers. The interpretation of the results will depend on the relative weight placed by decision-makers on clinical and economic outcomes. To readers focussed on clinical outcomes, the trial demonstrated that health coaching led to no changes in activation or quality of life. However, the economic analyses showed that the intervention was likely to represent a cost-effective use of resources at conventional levels of willingness to pay. The economic analysis examines the effect of health coaching using a generic measure of health-related quality of life, which may detect broader impacts of the intervention not captured by the primary trial outcomes. It also considers the trade-off between differences in costs and effects associated with the intervention.

Decision-makers may not be convinced of the benefits of health coaching in the absence of evidence of clinical improvement. However, resource utilisation patterns highlighted interesting results which warrant further investigation. Individuals offered health coaching had higher utilisation of planned services and lower use of emergency hospital services. Health coaching may have had a positive impact by increasing individuals’ wider engagement in the health service. Due to the limited follow-up period of the trial, we are not able to assess whether such increased engagement with planned services is maintained.

Health coaching in patients with multimorbidity did not lead to significant benefits on the primary measures of patient-reported outcome. The optimal role of this model of care within integrated care systems for patients with multiple long-term conditions remains unclear.

## Additional files

Additional file 1: CONSORT 2010 checklist of information to include when reporting a randomised trial. (DOC 218 kb)

Additional file 2: Protocol for the PROTECTS trial. (DOC 356 kb)

Additional file 3: **Table A.** Comparison of participants consenting with those not consenting. **Table B.** Costs of the health coaching intervention. **Table C.** Other NHS unit costs (XLSX 17 kb)

Additional file 4: The results of the cost-effectiveness analyses in complete case analysis. (DOCX 2606 kb)

## Abbreviations

CACE: Complier Average Causal Effect
CEACs: Cost-effectiveness acceptability curves
CLASSIC: Comprehensive Longitudinal Assessment of Salford’s Integrated Care
ICER: Incremental cost-effectiveness ratio
MHI-5: Mental Health Inventory
NHS: National Health Service
NICE: National Institute of Health and Clinical Excellence
PAM: Patient Activation Measure
PROTECTS: Proactive Telephone Coaching and Tailored Support
QALYs: Quality-adjusted life years
QOL: Quality of life
SDSCA: Summary of Self-Care Activities
TwiCs: ‘trial within a cohort’
WHOQoL-BREF: World Health Organization Quality of Life brief measure
