# Supplementary material for: Is telephone health coaching a useful population health strategy for supporting older people with multimorbidity? An evaluation of reach, effectiveness and cost-effectiveness using a ‘trial within a cohort’
Source: BMC Med. 2018 May 30;16:80. doi: 10.1186/s12916-018-1051-5 (PMC5975389; doi:10.1186/s12916-018-1051-5)
Supplement: Supplementary file 2 — Protocol for the PROTECTS trial. (DOC 356 kb) [file 12916_2018_1051_MOESM2_ESM.doc]

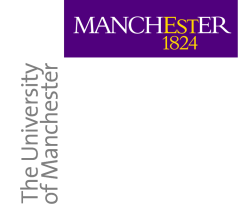


**CLASSIC Proactive Telephone Coaching and Tailored Support (PROTECTS)**

**A pragmatic, two-arm, patient-level, randomised trial of the effectiveness of telephone coaching and support for older people with multimorbidity**

| Trial registration |  | ISRCTN12286422 |
| --- | --- | --- |
| Protocol version |  | 1.1 |
| Funding |  | NIHR HSDR 12/130/33 |
| Roles and responsibilities |  | Principal investigator: Peter Bower  Statistician: Mark Hann  Design: Peter Coventry, David Reeves  Intervention development: Karina Lovell, Tom Blakeman, Waquas Waheed |
|  |  | Project manager: Mark Sidaway  Research staff: Kelly Howells  NHS collaborators: Charles Donnison, Christine Camacho |
|  |  | The funders will have no role in the collection, management, analysis, and interpretation of data; writing of the report; or the decision to submit the report for publication |
|  |  |  |

**Introduction**

*Background and rationale*

The burden of disease in Salford, the UK and most parts of the world is shifting to long-term conditions. Although significant advances have been made in effective service delivery, major challenges have created a ‘burning platform’ for change: projected increases in those aged 65+, increases in demand associated with an ageing population, and central government pressure for major efficiency savings (£20 billion by 2014/15).

Current services are organised around single long-term conditions, but many people have multiple conditions, which means that care is often fragmented and unresponsive to needs, with low levels of involvement.^1;2^ A raft of policy and patient consultation around long term conditions has repeatedly emphasised the need for integration, recommending that every person with long‐term or complex needs has access to a named contact who can coordinate care.^3;4^

The Salford Integrated Care Programme (SICP) is a large scale transformational project to improve care for older people with long-term conditions and social care needs in Salford, through 3 mechanisms:

(a) improved access to community resources and targeted support for self-management

(b) better integration of care through multidisciplinary health and social care groups

(c) an ‘Integrated contact centre’ to support navigation and self-management.

The contact centre will involve a number of services, which will provide support, navigation and other activities to support older patients in Salford. A key function is ‘health coaching’ for patients with long term conditions.

Health coaching involves ‘a regular series of phone calls between patient and health professional...to provide support and encouragement to the patient, and promote healthy behaviours such as treatment control, healthy diet, physical activity and mobility, rehabilitation, and good mental health’.^5^

Health coaching is designed to provide an accessible, proactive service to patients, improving support and potentially reducing inappropriate care utilisation. However, current evidence on the effectiveness of health coaching is mixed. Although systematic reviews have identified a number of effective models, outcomes are variable and it is not entirely clear what distinguishes effective models from those that fail to show effects. Some of the evaluations have used non-randomised designs which are vulnerable to bias.^6^ Many of the evaluations have been undertaken in the United States, and it is not clear that the results will generalise to populations with different characteristics, living in different contexts. A recent evaluation of the Birmingham OwnHealth service in 2698 patients and matched controls did not find reductions in health service utilisation associated with a nurse led health coaching service, although other outcomes (such as empowerment and quality of life) were not measured.^7^

Attempts to identify key drivers of effectiveness of health coaching have focussed on the content of the coaching (self-management versus ‘preference sensitive’ decision making), the types of coaches (health professionals versus paraprofessionals), the scope of the calls (such as whether mental health and substance abuse issues are dealt with alongside physical health and self-management) and the presence of additional interventions (such as telemonitoring).^8^

Two relevant evaluations have been conducted in services in the North West. The PACCTS study randomised 591 patients with Type 2 diabetes to telephone support from paraprofessionals, backed up by diabetes specialist nurses (the so-called Diabetes CareCall model). The study found reductions in HbA1c compared to usual care controls, with the most marked and clinically significant changes in those with poor glucose control at baseline.^9^

The BRIGHT study funded by the NIHR CLAHRC for Greater Manchester randomised 436 older patients with CKD to a telephone support intervention, conducted by paraprofessionals and supported by a bespoke website,^10^ and focussed on increasing patient access to health and other relevant resources in local networks. The study found that health coaching led to improvements in health related quality of life and blood pressure control, and was highly likely to be cost-effective.^11^

These effective local evaluations suggest that there is a role for health coaching interventions, although the pattern of results suggest that it is easier to improve quality and outcomes rather than reduce utilisation.^12^ The CLASSIC evaluation of health coaching will test a combination of the Diabetes CareCall and BRIGHT models.

The scope of the eligible populations will be widened to include patients with a wider range of conditions (with a focus on multimorbidity), given the known commonalities in challenges and self-management tasks among long-term conditions.^13;14^

In addition, mental health has great importance in populations with long-term conditions and has a significant impact on health care utilisation.^15;16^ The scope of the telephone coaching model will be expanded to include a greater focus on common mental health problems and low mood, building on our previous work in this area.^17;18^

**Objectives**

To test the effectiveness and cost-effectiveness of telephone coaching and support for older people with multiple long-term conditions.

*Trial design*

A pragmatic, two-arm, patient-level, randomised trial.

The study is a pragmatic effectiveness and cost-effectiveness trial, and thus the optimal comparator is a usual care control representing usual practice. The protocol is presented according to the SPIRIT guidelines.^19^

**Methods: Participants, interventions, and outcomes**

*Study setting*

Salford is in the North West of England, which has a population of approximately a quarter of a million (34,000 aged 65+) and contains around 50 practices, clustered in 8 neighbourhoods. The area has high levels of deprivation and long-term illness.

The Salford Integrated Care Programme (SICP) is a large scale transformational project to improve care for older people with long-term conditions and social care needs in Salford, through 3 mechanisms.

- Improved access to community resources and targeted support for self-management
- Better integration of care through multidisciplinary health and social care groups
- An ‘integrated contact centre’ to support navigation and self-management

The Comprehensive Longitudinal Assessment of Salford Integrated Care (CLASSIC) is an evaluation framework based on a cohort multiple randomised controlled trial,^20^ designed to provide a rigorous test of the ability of the SICP to deliver enhanced experience of care, improved outcomes and cost effectiveness.

The CLASSIC PROTECTS trial (Proactive Telephone Coaching and Tailored Support) is a pragmatic, individual level randomised trial to evaluate the effectiveness and cost-effectiveness of one part of the SICP – telephone coaching within the ‘Integrated contact centre’.

*Eligibility criteria*

All patients will be aged 65+, as this is the scope of the SICP.

For inclusion in CLASSIC PROTECTS, patients must have:

- 2 or more existing long-term conditions
- Assessed as needing some assistance with self-management (defined in terms of scores on the validated self-management scales in the cohort assessment). The exact range will be determined when initial data from the CLASSIC cohort are available.

**Interventions**

The intervention in CLASSIC PROTECTS is health coaching, defined as:

‘a regular series of phone calls between patient and health professional...to provide support and encouragement to the patient, and promote healthy behaviours such as treatment control, healthy diet, physical activity and mobility, rehabilitation, and good mental health’.^5^

We describe the intervention according to the TIDIER guidelines.^21^

*Why*

The health coaching intervention will be based on three core mechanisms:

- health coaching;
- social prescribing;
- low-intensity support for low mood

As noted, health coaching involves support and encouragement to the patient, to promote healthy behaviours, and involves 3 domains: information, ‘readiness to change’, and medication adherence.^9^

*Social prescribing* refers to linkage between health services for long-term conditions, and resources in the wider community through the community and voluntary sector.^22;23^

*Low-intensity support for low mood* includes simple assessment of common mental health problems, simple lifestyle advice and behavioural techniques to manage mood, and appropriate risk protocols.^24;25^

**Description of the intervention (SPIRIT guidelines)**

| *What* | *Telephone health coaching -* the core telephone and health coaching materials include telephone and associated patient tracking and management software, and health coaching scripts for lifestyle support around diet, exercise, smoking and alcohol) for long-term conditions.  *Social prescribing –* we will provide access to local resources in Salford, through either PLANS (<http://www.plansforyourhealth.org/> - a self-assessment tool for users to assess their health and social needs, with links to relevant community resources and local support) or the Salford Ways to Well-being site (https://www.way2wellbeing.org.uk/) . Where appropriate, the health coaching will guide participants through the sites to help them find resources.  *Support for low mood* - in managing older people with long-term conditions, telephone coaching staff are often managing patients with low mood. We will train health advisors will provide support for patients with low mood, around 3 core areas: assessment of the presence of symptoms; advice and behavioural activation; risk assessment. |
| --- | --- |
| *Who* | The PROTECTS intervention will be delivered by a health advisor (Agenda for Change band 4 worker) with essential skills in working with information technology and communication, as well as experience of working with the general public, good time management and an ability to work flexibly and under time pressure.  The health advisors will be supported by specialist nurses and managers within the Centre of Contact, with additional advice around mental health and social prescribing from the CLASSIC academic team. |
| *How and where* | The health coaching will be delivered via telephone from a central facility. The core interventions will be out-bound calls by the health coaching staff. The facility is Orbit House in Salford. |
| *When and how much* | Proactive, monthly calls of around 20 minutes will be made for a period of six months, with the option for additional calls to deal with complex patients or issues of risk. The main basis of the intervention will be outbound calls, although unscheduled inbound calls will be managed and recorded. |
| *Tailoring* | Health coaching staff will be trained to customize the pace and detail of the call to the social context of the individual patient. Provision of support for low mood, and access to community resources through PLANS will be made for those patients for whom it is relevant and appropriate. |
| *Modifications* | We do not envisage major changes to the delivery of the intervention through the study. |
| *How well* | The fidelity of the intervention will be assessed by qualitative work with patients and staff, as part of the broader CLASSIC research programme and the Implementation 2 theme. |

**Outcomes**

The CLASSIC study is based on a cohort multiple randomised controlled trial,^20^ where a large population cohort is recruited and followed systematically over time. The cohort provides an on-going assessment of the impact of the SICP changes over time, while subgroups of the cohort used to evaluate different interventions.^20^

In the cohort multiple randomised controlled trial, participants will be followed up every 6 months with brief measures of service experience, health and care outcomes and utilisation. Measures will be linked to routine data in the Salford Integrated Record and a social care dataset to provide data on clinical parameters, medication use, and interactions with NHS and social care services.

The cohort includes the following measures.

*(i) Baseline Demographic and clinical characteristics*

Baseline assessment includes:

- Socio-demographic questions from the General Practice Patient Survey,^26^ including gender, age, current work situation, and qualifications.
- Ethnicity assessed using the 17 Census 2011 categories.
- A single item health literacy measure which has demonstrated good reliability and validity^27;28^
- A measure of the number and impact of long-term conditions.^29^
- The ENRICHD measure of social support, a seven item scale with items on partners, tangible help and emotional support.^30^

The following measures will be used at baseline, and at all follow up points in the CLASSIC cohort.

*(ii) Health experience and self-management*

- PACIC. The *Patient Assessment of Chronic Illness Care (PACIC)* is a validated measure of patient assessments of service delivery for long-term conditions.^31;32^ The scale includes items in 5 subscales: patient activation; delivery system design; decision support; goal setting; problem-solving; and coordination.^32^ We use the short 11 item version.^33^
- PAM. The ***Patient Activation Measure* is a self-report measure of patient** knowledge, skills and confidence in self-management for long-term conditions.^34;35^ We use the short 13 item version. ^36^
- SDSCA. The Summary of Self-Care Activities (SDSCA) measure assesses the number of days per week respondents engage in healthy and unhealthy behaviours (i.e. eating fruit and vegetable, eating red meat, undertaking exercise, drinking alcohol, and smoking).^37^
- MULTIPLES. This scale assesses patient experience of managing multimorbidity.^38^ We will use 16 items from the MULTIPLES scale

*(iii) Health and social care outcomes*

- EQ5D. The five-item EQ-5D is a generic measure of health-related quality of life, suitable for people with long-term conditions and recommended by NICE for economic evaluations.^39^ The self-report measure consists of the EQ-5D descriptive system and EQ Visual Anologue Scale (EQ VAS). The first part consists of 5 items: mobility, self-management, usual activities, pain, anxiety and depression. Each dimension has three levels of severity and provides a utility value based on a population tariff.^40^ The VAS records an individual’s perceived self-rated health. We use the new EQ5D-5L version.
- MHI-5. The Mental Health Inventory (MHI-5) is a 5-item scale which measures general mental health, including depression, anxiety, behavioural-emotional control and general positive affect.^41;42^ This is complemented by a single item measure of ‘entrapment’.^43^
- ICECAP-O. The ICECAP-O index of capability measures quality of life for people aged 65 or over in terms of 5 core dimensions: attachment, security, role, enjoyment and control. It is anchored at 0, for no capability, and 1 for full capability.^44;45^
- WHOQoL-BREF. The World Health Organization Quality of Life brief measure (WHOQoL-BREF) is a 26-item measure of global QOL, which has been validated in a large international population with physical and mental long term conditions. QOL is measured across four domains: physical, psychological, social, and environmental, as well as a single-item scale for QOL.^46^
- We use items on health care utilisation, based on our previous CAPITOL^47^ and CHOICE studies (<http://choice.mhsc.nhs.uk/home.aspx>), an item on difficulties in accessing health care from the Canadian Community Health Survey,^48^ and an item on use of community resources from the Health Survey for England,^49^, as well as items on continuity of care and care planning from the General Practice Patient Survey.^26^ We also include items on patient experience of safety from the integrated care pilots evaluation.^50^
- We will use bespoke measures assessing use of email communication around health care, and three items assessing issues of interest to stakeholders in the SICP.

We will include a shorter assessment for carers of those identified in the main CLASSIC cohort, including EQ5D, PHQ 9, ICECAP-O and the Modified Caregiver Strain Index used in our recent assessment of the Whole System Demonstrators.^51^

*(iv) Care utilisation and costs*

We will obtain information on rates of utilisation of most of the major elements of health and social care through linkage to the Salford Integrated Record. These will be costed using national tariffs or published unit costs. We will also include short questions taken from previous evaluations of WISE and the EPP on patient out-of-pocket payments, time spent on self-care, and amount of informal care received from personal social networks.

The primary outcomes for CLASSIC PROTECTS are:

(i) Health experience and self-management

The Patient Activation Measure (PAM)

(ii) Quality of life

The World Health Organization Quality of Life brief measure (WHOQoL-BREF)

(iii) Cost effectiveness

EQ5D and measures of health care utilisation

*Participant timeline*

Participants will be in the CLASSIC cohort for around 2 years, with assessments every six months.

*Sample size*

We powered the study to have 80% power (with an alpha level of 5%) to detect a standardised effect size of 0.25 between the control and intervention arms on any continuous outcome measure. Allowing for 25% attrition amongst participants – and treating outcome measures at baseline as covariates which correlate at, conservatively, 0.5 with their respective follow-up measurements, 504 patients will need to be recruited at baseline (252 per trial arm). These numbers will also suffice to provide 80% power to detect a difference as small as 15% between the two trial arms on any binary outcome measure.

*Recruitment*

The CLASSIC study is based on a cohort multiple randomised controlled trial,^20^ where a large population cohort is recruited and followed systematically over time. As part of the cohort, participants will be followed up every 6 months with brief measures of service experience, health and care outcomes and utilisation. Measures will be linked to routine data in the Salford Integrated Record and a social care dataset to provide data on clinical parameters, medication use, and interactions with NHS and social care services.

As part of the cohort, we seek consent to contact people about further CLASSIC sub studies, including CLASSIC PROTECTS. The cohort will facilitate recruitment by allowing proactive contact with respondents.

If recruitment to CLASSIC PROTECTS through the cohort is insufficient, we will bolster recruitment with eligible older people recruited through other forms, such as self-referral and referral through the ‘Integrated Contact Centre’, through primary care, or post discharge referral from specialist settings.

**Methods: Assignment of interventions (for controlled trials)**

Participants will be enrolled through the CLASSIC cohort, and other sources. After assessment of eligibility and informed consent, we will allocate patients randomly to ‘health coaching’ or usual care using appropriate central randomisation through the accredited Manchester Academic Health Science Centre (MAHSC) clinical trials unit to ensure concealment of allocation.

In this pragmatic, health service evaluation, there is no blinding of patients or providers. All outcomes are either self-report or using routine data.

*Data collection*

CLASSIC PROTECTS will function within the CLASSIC cohort study, and we expect that the bulk of patients randomised will be identified from the cohort.

Assessments will use a combination of telephone and postal administration, with appropriate support to enhance response rates and maximise internal and external validity. We have extensive experience of the delivery of large scale surveys among people with long-term conditions and the appropriate mix of postal surveys and telephone support, and we have costed for travel for face to face visits to support frail respondents, and intensive telephone support for those who require more limited assistance. In recent studies (including Salford samples) we have recruited large samples of 2000-5000 patients with long-term conditions and achieved good retention rates over 12 months.^32;52^

Data will be double entered to ensure accuracy, and stored on secure University servers.

*Data analysis*

We will report the trial and analysis according to updated CONSORT standards,^53^ including full details of use of the various telephone coaching components, linking to analysis of patient data in Implementation 2 to contextualise the quantitative findings. We will utilise the extension for pragmatic trials.^54^

Analysis will follow intention to treat principles and a pre-specified analysis plan. Binary outcomes will be analysed using logistic regression and continuous outcomes using linear regression, controlling, where appropriate, for baseline values of the respective outcome. Due to the longitudinal cohort design of the CLASSIC study within which PROTECT is nested, individual patient baseline and follow-up outcome measurement times may differ from the notional PROTECT study times by up to 6 months. This will be accounted for in the analysis through the use of covariates to control for trends in outcomes over these periods. We will also examine ways of increasing power by inclusion of multiple baseline and follow-up measurements for those patients who have them. All analyses will use either robust or, for highly skewed data, bootstrapped (based on 1,000 samples) standard error estimation and will adjust for the clustering of patients within practices. Multiple imputation, using chained equations, will be used to impute missing values for all outcome measures.

**Monitoring**

We will set up an appropriate trial steering committee to oversee conduct and progress of the trial, including representation from clinical, statistical and patient representatives.

The CLASSIC PROTECTS study is based on modification of an existing service, where the likely risks are minimal, and we do not envisage that analysis of interim data would lead to protocol modification. Therefore, a data monitoring committee (DMC) will not be used.^55^ We do not plan any interim analyses or stopping rules.

A formal application for ethical approval and research governance will be made. All details concerning consent, confidentiality, access to data and other ethical issues will be described in full in the application

*Consent*

Patients will already have consented to the CLASSIC cohort, and we will use the cohort as the main source of patients for CLASSIC PROTECTS.

CLASSIC PROTECTS uses a cohort multiple randomised controlled trial design.^20^ There are two special features of this design:

1. Cohort recruitment

First, an existing cohort is used to recruit patients to the trial. We already have ethical approval for the CLASSIC cohort. All patients in the CLASSIC cohort will have provided details of their personal and clinical characteristics. We will use that data to identify patients who are eligible for the CLASSIC PROTECTS study

2. Patient centred consent and randomisation procedures

In a standard trial informed consent procedure, patients receive information about the trial and the interventions available, and then provide written informed consent to take part in the trial. At that point, they are randomised between the trial arms - often between a new intervention we want to test, and usual NHS care (see Appendix 1 Option 1).

A significant drawback of this design is that patients are told about different treatments in the different arms, including any new treatment, but only half the patients are randomised to that new treatment. This can cause disappointment and dissatisfaction in patients.

This procedure is also very different to usual NHS treatment, where people are NOT told about treatments that they cannot access. They are only asked to consent to treatments that they are being offered.

The ‘cohort multiple randomised controlled trial design’ takes a different approach to avoid these problems, and tries to make the procedure more like standard NHS care.

Patients who are potentially eligible for the trial are identified from the cohort, and randomly selected. Patients who are randomised to usual care simply continue to be followed up in the cohort, and are not informed about the trial or the randomisation. This avoids telling patients about treatments they will not receive.

Patients who are randomised to the new treatment are then contacted and offered the treatment. They still provide consent to the new treatment, and can decide whether they wish to receive it.

If patients agree to the new treatment, they are provided with the new treatment, and continue to be followed up in the cohort. If patients decide that they do not wish to receive the new treatment, they continue to receive usual care, and continue to be followed up in the cohort (see Appendix 1 Option 2).

The ‘cohort multiple randomised controlled trial design’ has been assessed as valid through peer review, but experience with the design is limited. There are some possible drawbacks of the design. If few patients randomised to the intervention group decide to take up the new treatment, the trial may not be able to provide a good test of the treatment.

Also, if not enough patients can be recruited through the cohort, we may need to use other recruitment procedures outside the cohort. However, if patients are recruited outside the cohort, they cannot be recruited using the ‘cohort multiple randomised controlled trial design’. Recruitment would need to use standard trial informed consent (see Appendix 1 Option 3).

CLASSIC PROTECTS does not have to use the ‘cohort multiple randomised controlled trial design’ recruitment procedure.

We will pilot these procedures in 50 patients to test the rate of uptake of the new treatment in CLASSIC PROTECTS. If the rate of treatment uptake is high enough (>60%) and we can recruit enough patients through the CLASSIC cohort, we will use the ‘cohort multiple randomised controlled trial design’ procedures throughout the CLASSIC PROTECTS trial (Option 2).

If the rate of treatment uptake is low, we will adopt standard trial informed consent procedures (Option 1) for all patients recruited beyond the first 50.

If we cannot recruit enough patients from the cohort alone, we will adopt standard trial consent procedures to recruit through other methods, such as recruitment via GPs, via post-discharge referrals, and through other sources within Salford (Option 3).

We will clearly describe the patients recruited by each method in our research reports. Our statistical analysis plan will make appropriate adjustments to take account of any differences between patients recruited by different methods.

*Dissemination policy*

We will disseminate the results of relevant studies in a range of academic journals, including general medical and health services research journals, as well as specialist policy, economics, informatics and mental health publications, through open access publication where possible.

*Declaration of interests*

None of the authors of the protocol have competing interests

**CLASSIC PROTECTS recruitment and consent procedure – OPTIONS FOR REC**

**Patients in the CLASSIC cohort**

**CLASSIC PROTECTS eligibility criteria**

**Patients eligible for CLASSIC PROTECTS**

**Random allocation**

**Health coaching**

**ALL patients in followed up in the CLASSIC cohort**

**Usual care**

**Informed consent to trial**

*Option 1 - Standard trial informed consent (inside CLASSIC cohort)*

*Option 2 - Cohort multiple randomised controlled trial patient centred consent (inside CLASSIC cohort)*

**Patients in the CLASSIC cohort**

**CLASSIC PROTECTS eligibility criteria**

**Patients eligible for CLASSIC PROTECTS**

**Random selection**

**Informed consent to receive intervention**

**Usual care**

**YES – health coaching**

**ALL patients in followed up in the CLASSIC cohort**

**NO- usual care**

*Option 3 - Standard trial informed consent (other recruitment methods)*

**Other recruitment sources**

**CLASSIC PROTECTS eligibility criteria**

**Patients eligible for CLASSIC PROTECTS**

**Random allocation**

**Health coaching**

**Follow up 6 months from random allocation**

**Usual care**

**Informed consent to trial**
