# Supplementary material for: Is telephone health coaching a useful population health strategy for supporting older people with multimorbidity? An evaluation of reach, effectiveness and cost-effectiveness using a ‘trial within a cohort’
Source: BMC Med. 2018 May 30;16:80. doi: 10.1186/s12916-018-1051-5 (PMC5975389; doi:10.1186/s12916-018-1051-5)
Supplement: Supplementary file 4 — The results of the cost-effectiveness analyses in complete case analysis. (DOCX 2606 kb) [file 12916_2018_1051_MOESM4_ESM.docx]

*Cost effectiveness analysis: Complete case analysis*

Table B presents the adjusted estimates of the effect of the offer of health coaching on the incremental costs and QALYs over and above usual care in the complete case sample.

Health coaching is associated with a mean incremental cost of £497.99 (95% CI: £-189.19, £1,185.19). Whilst higher than the incremental cost associated with health coaching in the full sample with imputation, this difference in cost between arms still does not reach statistical significance. The incremental QALY estimate is also higher 0.037 (95% CI: 0.0037, 0.070), indicating that there was a significant QALY gain associated with health coaching amongst the complete case sample. This results in an ICER of £13,506.27 per QALY, which is again below the lower bound of recommended cost-effectiveness thresholds. It is again important to examine the uncertainty around these point estimates in probabilistic terms.

Figure A shows that the bootstrapped replications are again clustered predominantly in the north-west quadrant. There is notable shift north above the x axis and to the right of the y axis compared to the full sample with imputation, as even more of the replications now fall into this quadrant, representing incremental increases in both costs and QALYs above usual care. Health coaching was more costly in 93% of replications, and lead to an incremental QALY gain in 99% of replications.

The CEAC illustrates that at the lower bound (£20,000 per QALY), there is a 67% probability that P health coaching is cost-effective, rising to 82% at the upper bound of £30,000 (Figure B). Compared with usual care, health coaching is likely to be cost-effective in 50% or more cases if decision makers are willing to pay £13,570 or more for one QALY.

**Table B: Cost-effectiveness analysis results: complete case sample**

| **Health coaching (n=206) over usual care (n=378)** | **Mean** | **Bootstrapped standard error** | **Bootstrapped 95% CI** | |
| --- | --- | --- | --- | --- |
| Incremental cost (£) | 497.998 | 350.6141 | -189.193 | 1185.189 |
| Incremental QALYs | 0.037 | 0.016947 | 0.003656 | 0.070088 |
| **ICER** | **£13,506.27** |  |  |  |

**Figure A. Cost-effectiveness plane: complete case sample**

**Figure B. Cost-effectiveness acceptability curve: complete case sample**

**Figure C. 0-6 month Cost-effectiveness plane full sample with imputation**

**Figure D. 0-6 month Cost-effectiveness acceptability curve: full sample with imputation**

**Figure E. 6-18 months Cost-effectiveness plane: full sample with imputation**

**Figure F. 6-18 months Cost-effectiveness acceptability curve: full sample with imputation**
